# Cholangiocyte Epithelial to Mesenchymal Transition (EMT) is a potential molecular mechanism driving ischemic cholangiopathy in liver transplantation

**DOI:** 10.1371/journal.pone.0246978

**Published:** 2021-07-07

**Authors:** Niluka Wickramaratne, Ru Li, Tao Tian, Jad Khoraki, Hae Sung Kang, Courtney Chmielewski, Jerry Maitland, Loren K. Liebrecht, Ria Fyffe-Freil, Susanne Lyra Lindell, Martin J. Mangino

**Affiliations:** 1 Department of Surgery, School of Medicine, Virginia Commonwealth University, Richmond, VA, United States of America; 2 Department of Physiology and Biophysics, School of Medicine, Virginia Commonwealth University, Richmond, VA, United States of America; 3 Department of Pharmacology and Toxicology, School of Medicine, Virginia Commonwealth University, Richmond, VA, United States of America; 4 Department of Emergency Medicine, School of Medicine, Virginia Commonwealth University, Richmond, VA, United States of America; Texas A&M University, UNITED STATES

## Abstract

Donation after circulatory death (DCD) has expanded the donor pool for liver transplantation. However, ischemic cholangiopathy (IC) after DCD liver transplantation causes inferior outcomes. The molecular mechanisms of IC are currently unknown but may depend on ischemia-induced genetic reprograming of the biliary epithelium to mesenchymal-like cells. The main objective of this study was to determine if cholangiocytes undergo epithelial to mesenchymal transition (EMT) after exposure to DCD conditions and if this causally contributes to the phenotype of IC. Human cholangiocyte cultures were exposed to periods of warm and cold ischemia to mimic DCD liver donation. EMT was tested by assays of cell migration, cell morphology, and differential gene expression. Transplantation of syngeneic rat livers recovered under DCD conditions were evaluated for EMT changes by immunohistochemistry. Human cholangiocytes exposed to DCD conditions displayed migratory behavior and gene expression patterns consistent with EMT. E-cadherin and CK-7 expressions fell while N-cadherin, vimentin, TGF_β_, and SNAIL rose, starting 24 hours and peaking 1–3 weeks after exposure. Cholangiocyte morphology changed from cuboidal (epithelial) before to spindle shaped (mesenchymal) a week after ischemia. These changes were blocked by pretreating cells with the Transforming Growth Factor beta (TGF_β_) receptor antagonist Galunisertib (1 μM). Finally, rats with liver isografts cold stored for 20 hours in UW solution and exposed to warm ischemia (30 minutes) at recovery had elevated plasma bilirubin 1 week after transplantation and the liver tissue showed immunohistochemical evidence of early cholangiocyte EMT. Our findings show EMT occurs after exposure of human cholangiocytes to DCD conditions, which may be initiated by upstream signaling from autocrine derived TGFβ to cause mesenchymal specific morphological and migratory changes.

## Introduction

Liver transplantation is the only potentially curative treatment for patients with end stage liver disease. However, due to the continued shortage of organ donors, only 50 liver transplants per 100 patient-years of wait time are performed annually in the United States resulting in significant pre-transplant waitlist mortality. In 2017, 1334 patients died while waiting for a liver and 1177 were removed from the wait list for becoming “too sick to transplant” [1]. In order to address this gap, utilization of organs from donation after circulatory death (DCD) is an important strategy to widen the donor pool. Organs procured from DCD donors are inherently exposed to warm ischemia that occurs between the time the donor’s heart stops beating and the time the liver can be flushed with cold preservation solution.

Relative to donation after brain death (DBD), DCD grafts have a 4-fold increased odds of organ discard with livers recovered but not transplanted (1). Nevertheless, the increased warm ischemia time (WIT) with DCD transplants leads to significantly lower graft and recipient survival rates compared to DBD. This is due to higher rates of early allograft dysfunction, acute organ failure, and severe biliary complications (2–5). Ischemic cholangiopathy (IC), defined as non-anastomotic strictures of the intra- or extrahepatic biliary tree in the presence of a patent hepatic artery [2–4], is the most common serious biliary complication. It has been reported in 4–12% and up to 34% within 6 and 12 months of DCD transplantation, respectively [5]. When compared to DBD, a meta-analysis found a 10.8-fold increased odds of IC in DCD livers [3]. IC is a particularly feared complication of liver transplantation because it can cause biliary sepsis, requiring multiple hospitalizations for invasive diagnostic and therapeutic interventions and eventually require re-transplantation, all of which ultimately reduce patient and graft survival [6–9].

While the etiology of IC is likely multifactorial, causative factors include ischemic events in the peri-transplant period, bile salt toxicity, and immune-mediated injury [10]. The ischemia during preservation of the donor liver and subsequent reoxygenation causes an ischemia-reperfusion injury (IRI) to which the biliary epithelium is especially sensitive [11]. The IRI results in loss of the biliary epithelium and subsequent fibrosis and narrowing of the bile ducts [10]. The presentation and clinical course of IC is heterogeneous and may lend itself to less invasive interventions such as stenting and dilation of the biliary tree, depending on the location of the strictures. However, in many cases, the only viable solution is re-transplantation [12, 13].

Because of its significant impact on post-transplant outcomes, IC has been called the Achilles heel of DCD liver transplantation, limiting the maximal utilization of livers from DCD donors. Although there are several techniques to minimize the risk of IC, including reducing ischemia times, thrombolytic therapy, and machine perfusion [14–16], little is still known about the cellular processes underlying its development. Elucidating the molecular mechanisms that drive the development of cholangiopathy may provide new therapeutic targets and biomarkers, thus creating avenues to optimize and expand the use of DCD donors.

Epithelial to mesenchymal transition (EMT), a cellular phenomenon that results in tissue fibrosis, may have a role in the pathogenesis of IC [17]. During EMT, cells lose their differentiated epithelial traits and gain a mesenchymal phenotype. The hallmarks of EMT include a loss of epithelial adhesions and polarity, a change in morphology to a fibroblast like shape, a gain in mesenchymal markers and an acquired ability to penetrate and migrate through the extracellular matrix [18]. Several triggers are known to cause EMT, including inflammation and hypoxia. EMT has been implicated in several fibrotic disorders. Specifically, evidence of EMT has been observed in biliary atresia, primary sclerosing cholangitis, and in early recurrent primary biliary cirrhosis after liver transplant [19–21]. Furthermore, EMT is a mediator of interstitial fibrosis and tubular atrophy after kidney transplant, which is one of the major causes of graft loss and is, in part, due to long ischemic times [22–24].

Given that EMT is associated with a loss of epithelial cells in response to IRI as well as a pro-fibrotic phenotype, it is reasonable to conclude that EMT may be involved in the development of IC. However, data regarding hypoxia-induced EMT in biliary epithelial cells is lacking. Thus, the goal of this study was to demonstrate the presence of an IRI-induced EMT in an in vitro model of preservation injury using primary human cholangiocytes. We hypothesize that cells exposed to ischemia and reperfusion will undergo an epithelial-to-mesenchymal transition, and that this effect will be more pronounced in cholangiocytes that are subjected to warm ischemia compared to cold storage alone. The long-term goal is to understand mechanisms of ischemic cholangiopathy after DCD liver transplantation to later serve as potential therapeutic targets to expand the liver donation pool and improve transplantation success. The data presented in this study were successful in demonstrating strong associations of EMT attributes (gene expression, morphology, cytokinesis) with known initiators of IC in DCD liver transplantation (prolonged preservation ischemia). We also demonstrate a causal link between TGF_β_ receptor activation and ischemia-induced cholangiocyte morphological changes characteristic of EMT. This holds promise for the goal of therapeutically targeting EMT circuits to prevent or reverse ischemic cholangiopathy in patients receiving an expanded criteria liver allograft.

## Materials and methods

### Cell model

Human primary biliary epithelial cells (cholangiocytes, Celprogen; Torrance, CA; catalog # 36755–12) were cultured on collagen plates using Human Cholangiocyte growth media (Celprogen). Cells at 80% confluency were used for studies. To simulate DCD recovery and preservation conditions, cell cultures were subjected to both periods of warm and cold ischemia. Cell culture plates were placed in a plastic airtight box (Tupperware) purged with 95% nitrogen and 5% CO_2_ for 5 minutes followed by placing the box first in a 37 degree water bath for 60 minutes and then in a cold room on melting ice for cold storage at 2–4 C for 20 hours. After storage, the cells were returned to the 37 C incubator, exposed to atmospheric oxygen, and cultured as usual. Cells were removed from culture at 1, 5, and 7 days after storage ischemia and reoxygenation for study and analysis. Some cells were pretreated with the selective TGFβ receptor antagonist Galunisertib in DMSO (1μM) or DMSO (0.03%) for an hour prior to exposure to ischemia.

### Cell migration assay

To both measure migratory and invasive behavior and to select out cells with such behavior, normal or preserved cholangiocytes were placed on 24-well cell invasion assay plates coated with collagen (CBA-110-Col, Cell Biolabs, San Diego, CA) and allowed to culture for 48 hours. After culture, cells that may have migrated through the collagen matrix and attached onto the fiber pad below were recovered, washed, and expanded in cell culture to select out specific migratory phenotypes for later analysis. Cells on the top that refuse to migrate were also collected and expanded in cell culture to serve as non-reactive controls. Finally, Naïve colangiocytes were also used as non-stimulated controls since they never experienced DCD conditions nor did they migrate through the collagen migration chambers.

### Cell morphology

Morphology of cultured cholangiocytes was assessed using light microscope at 400x magnification in normal (Naive) cells before and after exposure to 24 hours of cold storage (CS) with or without 1 hour of warm ischemia prior to the CS. Cell morphology is graded on a scale of 1–4 in a blinded manner. A score of (1) refers to normal cuboidal appearing epithelial cells, (2) are mostly cuboidal appearing cells, (3) refers to most cells having a spindle appearing mesenchymal cell morphology, and (4) refers to all cells having a spindle mesenchymal appearance.

### Immunocytochemistry

Cells were grown on cover slips, fixed in 10% formalin, permeabilized with citrate buffer microwaving, and stained with specific primary antibodies to one of the following targets: cytokeratin-7 (CK-7), E-Cadherin, SNAIL, N-Cadherin, and Vimentin. After incubating for 2 hours at 37 C, the primary antibodies were washed away and the cells were probed with a secondary antibody directed at the primary and labeled with Alexa Fluor-488 or -647. Imaging was performed using a fluorescent microscope and images processed using ImageJ (NIH, Bethesda, MD, USA). These analyses were performed and compared between 3 groups of cholangiocytes: normal control cholangiocytes, (Fresh), cells exposed to 24 hours of cold ischemia storage (CS) and cells exposed to 60 minutes of warm ischemia conditions followed by 24 hours of cold ischemia storage (WI+CS). Analyses were repeated for cells cultured for 24 hours, and 1, 2 and 3 weeks after the corresponding storage conditions.

### Reverse transcription polymerase chain reaction

For the initial RNA isolation procedures, the RNeasy Mini Kit (Qiagen, Germantown, MD) was utilized according to manufacturer’s instructions. Briefly, 1 x 107 cells were pelleted and lysed with RLT buffer from the kit. 70% ethanol was added, the sample was transferred to the RNeasy Mini spin column, and the column centrifuged for 15 seconds at > 10,000 rpm. After several column wash steps, the RNA was eluted from the column membrane with 30 μl RNase-free water. Residual DNA contamination was removed by a DNase treatment protocol (Qiagen RNase-free DNase Set). Purified RNA was analyzed on the NanoDrop ONE for purity and concentration.

Purified, quantitated RNA samples were converted into complementary DNA (cDNA) for compatibility with subsequent qRT-PCR. A commercially available reverse transcriptase (RT) cDNA synthesis kit (iScript cDNA Synthesis Kit, Bio-Rad) was used on RNA samples, producing a presumed 1:1 cDNA product.

A CFX Connect Real-Time PCR Detection System (Bio-Rad) was used for real-Time PCR. cDNA samples were transferred to a 96-well PCR plate and iTaq Universal SYBR Green Supermix (Bio-Rad), and forward / reverse primer pairs for human TGF_β_ were added to each well. The plate was sealed, centrifuged for 5 minutes on a plate-spinner, and then analyzed in the CFX Connect according to manufacturer’s instructions. CFX Maestro software collects, compiles, and analyzes the resulting data, including amplification cycle and melting curves. Data were expressed relative to the GAPDH reference product.

### Rat liver transplant model

With the approval of the VCU Institutional Animal Care and Use Committee (IACUC), syngeneic liver transplants between adult male Brown Norway rats (Envigo, North America, n = 24) were performed as previously described by our group [25, 26]. All rats were housed in single cages in the VCU Vivarium and were allowed food and water ad libitum before surgery and after. After surgery, the rats were cared for in the lab for 24 hours to ensure wound closure, infection control, and hemostasis. Then they were returned to the vivarium for another 6 days with daily monitoring by lab personnel and staff veterinarians. All rats were anesthetized and maintained in a surgical plane of anesthesia with inhalation isoflurane (1–2%) during the surgery with 100% oxygen as the carrier gas. Some donor livers were subjected to a 30-minute warm ischemia period prior to recovery by clamping the hepatic pedicle (DCD conditions) and all were flushed with cold UW solution and stored for 20 hours at 2–4 C before transplantation. Donors died of exsanguination under anesthesia during the liver recovery. Recipient livers were removed through a midline incision. The inferior vena cava, portal vein, and bile duct were cuffed with PE tubing sections and tied into the respective recipient vessels with 7–0 proline. The superior vena cava was anastomosed with 8–0 proline and the livers were revascularized within 18 minutes from the start. After surgery, rats were allowed to awaken after receiving buprenorphine SR Lab for post-operative pain control for 72 hours. Recipient rats were monitored for vital signs every 15 minutes until upright and then every hour for the next 4 hours. Animal well-being was assessed by the presence of normal physiological variables and the absence of pathological indicators such as infection, refractory pain, or porphyrin staining. Two-ml of warm lactated Ringers solution was administered subcutaneous for volume replacement. Serial measurements of alanine aminotransferase (ALT) and total bilirubin were obtained 1, 3 and 7 days after surgery by penile vein blood draw (0.2 ml). One week following transplantation and at the onset of hyperbilirubinemia in the DCD liver recipients, the rats were re-anesthetized with isoflurane and the liver grafts removed for immunohistochemical (IHC) evaluation of SNAIL and CK-7 using HRP-labelled primary antibodies against rat SNAIL and CK-7 followed by staining with DAB. Recipient rats were euthanized by exsanguination under anesthesia during the recovery of the livers grafts.

### Statistical analyses

All data were tested for distribution normality. Some data were analyzed by parametric One-way ANOVA with Bonferroni multiple comparison correction. Cell counts, histological scores, and relative intensity staining or expression ratios were analyzed by the non-parametric Kruskal-Wallis one-way ANOVA followed by the Mann-Whitney test for stochastic dominance. Most data are expressed as mean plus or minus the standard deviation. IHC and other cell results were derived from 4 independent experiments usually run in triplicate. Liver transplants were repeated 6 times per group. Statistical analysis were performed using GraphPad InState and Prism software. A P value less than 0.05 was considered statistically significant.

## Results

### Human cholangiocytes cell model

**Fig 1:**
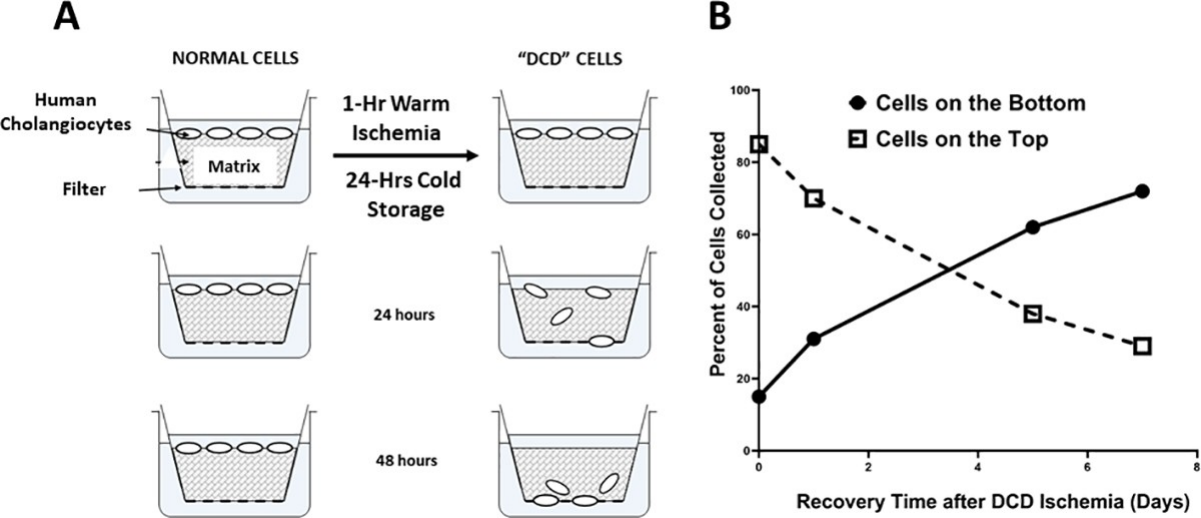
Cholangiocyte migration assay. Cholangiocyte migration was measured in an invasion chamber using a collagen gel matrix to detect cells with invasive and migratory behavior after exposure to DCD conditions (panel A). Cholangiocytes migrated through the matrix at increased rates depending on how long they were allowed to recover after exposure to DCD conditions. Specifically, a progressively larger percentage of cells reached the bottom of the chamber after 48 hours as the recovery time increased from 1–7 days (panel B). Results are expressed as mean +/- SD, n = 5.

Cholangiocytes exposed to DCD conditions (DCD Cells) had a significantly increased migratory behavior on the cell invasion assay as shown in **Fig 1**. This was demonstrated by significantly higher cell staining of cells being measured on the fiber pads under the collagen matrix, indicating cells from above physically moved from top to bottom through the matrix. Fresh control cholangiocytes without exposure to DCD conditions (Normal Cells) did not migrate to reach the lower fiber pad.

**Fig 2:**
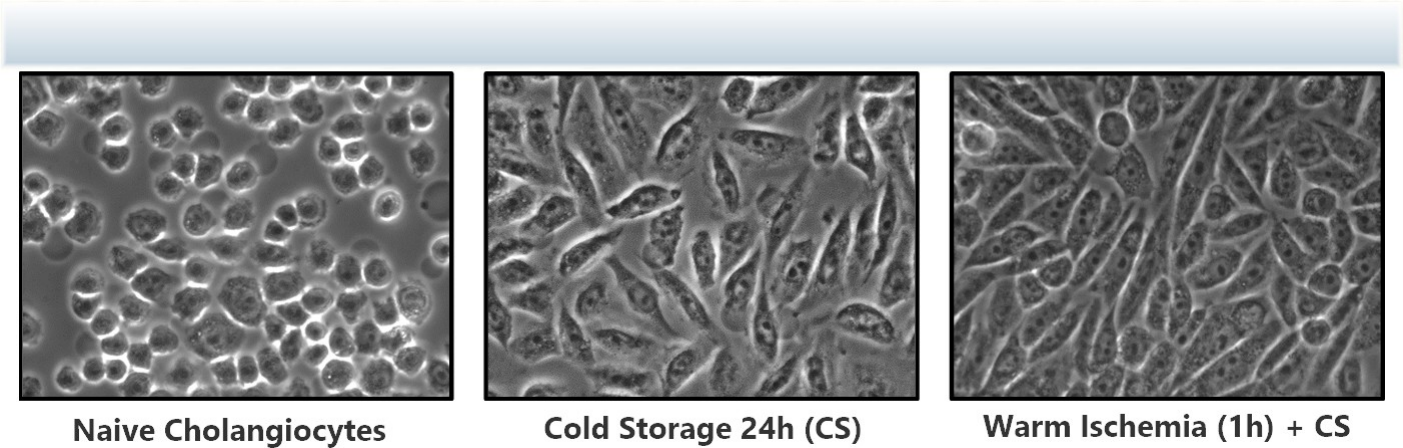
Cholangiocyte morphology changes. Light microscopic appearance of cultured cholangiocytes before or after cold storage (24 hours) and warm ischemia (1 hour). Panel 1 shows Naive cholangiocytes: Normal cuboidal epithelial cell appearance; Panel 2 shows 24-hour cold storage cells: Mixed cell shapes from cuboidal towards spindle-shaped cell; Panel 3 shows cells exposed to both cold storage and 1 hour of warm ischemia: Mostly spindle mesenchymal cell appearance with loss of cuboidal architecture in any cells. The normal cuboidal epithelial appearance has been replaced by a spindle shaped mesenchymal appearing cell morphology in response to DCD ischemic conditions. Magnification = 400x.

Cells that were exposed to prolonged cold storage for 24 hours with or without prior warm ischemia for one hour displayed a mesenchymal morphology compared to their baseline cuboidal epithelial cell appearance. These morphological changes are clearly seen in **Fig 2** with DCD cells exposed to both WI and CS expressing morphology similar to mesenchymal cells.

**Fig 3:**
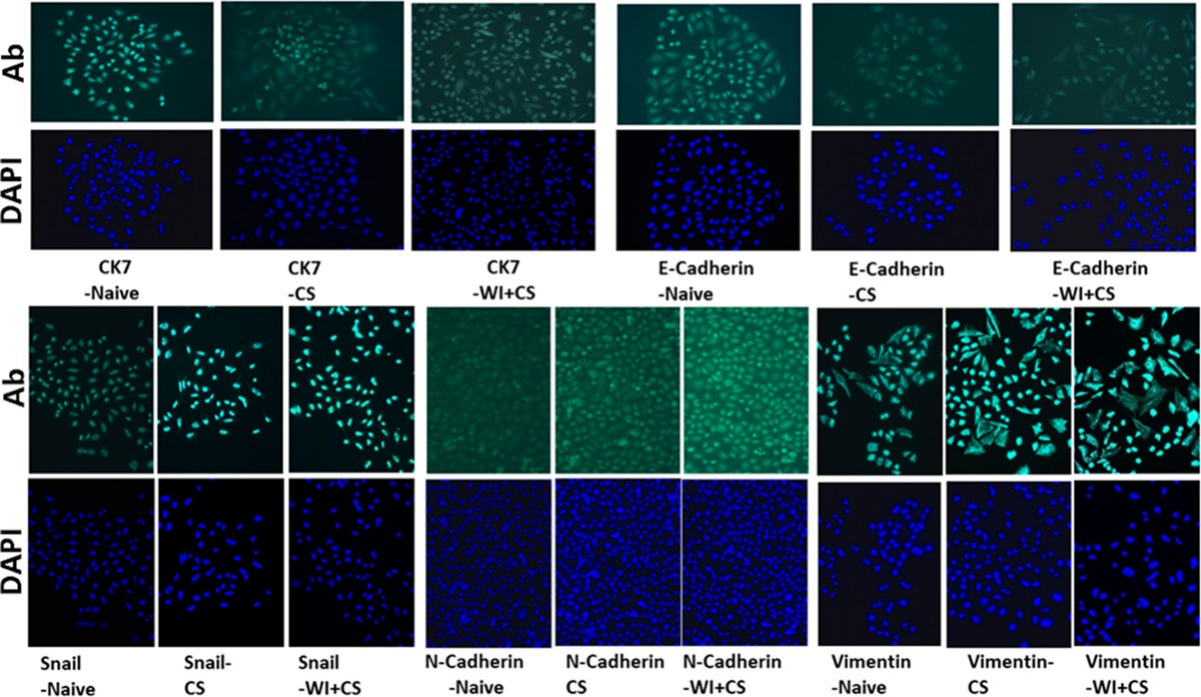
Immunocytochemical (ICC) staining of the epithelial cell markers. Human cholangiocyte-specific protein staining (upper panels) includes both CK7 and E-cadherin from groups of cells that include Naïve cells without DCD ischemia, from cells with 24 hour exposure to cold storage (CS), and from cells exposed to both cold storage and 60 minutes of warm ischemia (WI). Each antibody (Ab) specific stain is paired with the same cells stained with the nuclear stain DAPI (blue images). For the epithelial markers, the magnitude of the specific staining DECREASES as the degree of DCD ischemia increases (Naïve to CS to CS+WI). The bottom groups of cells are similar to the top except these cells were stained for the mesenchymal specific markers SNAIL, N-cadherin, and Vimentin. Opposite to the pattern seen with epithelial markers, the mesenchymal specific markers are shown to INCREASE in staining intensity as the level of DCD ischemic stress increases (Naïve to CS to CS+WI). These protein expression patterns are consistent with cells undergoing an epithelial-to-mesenchymal transition (EMT). Magnification is 200x.

**Fig 4:**
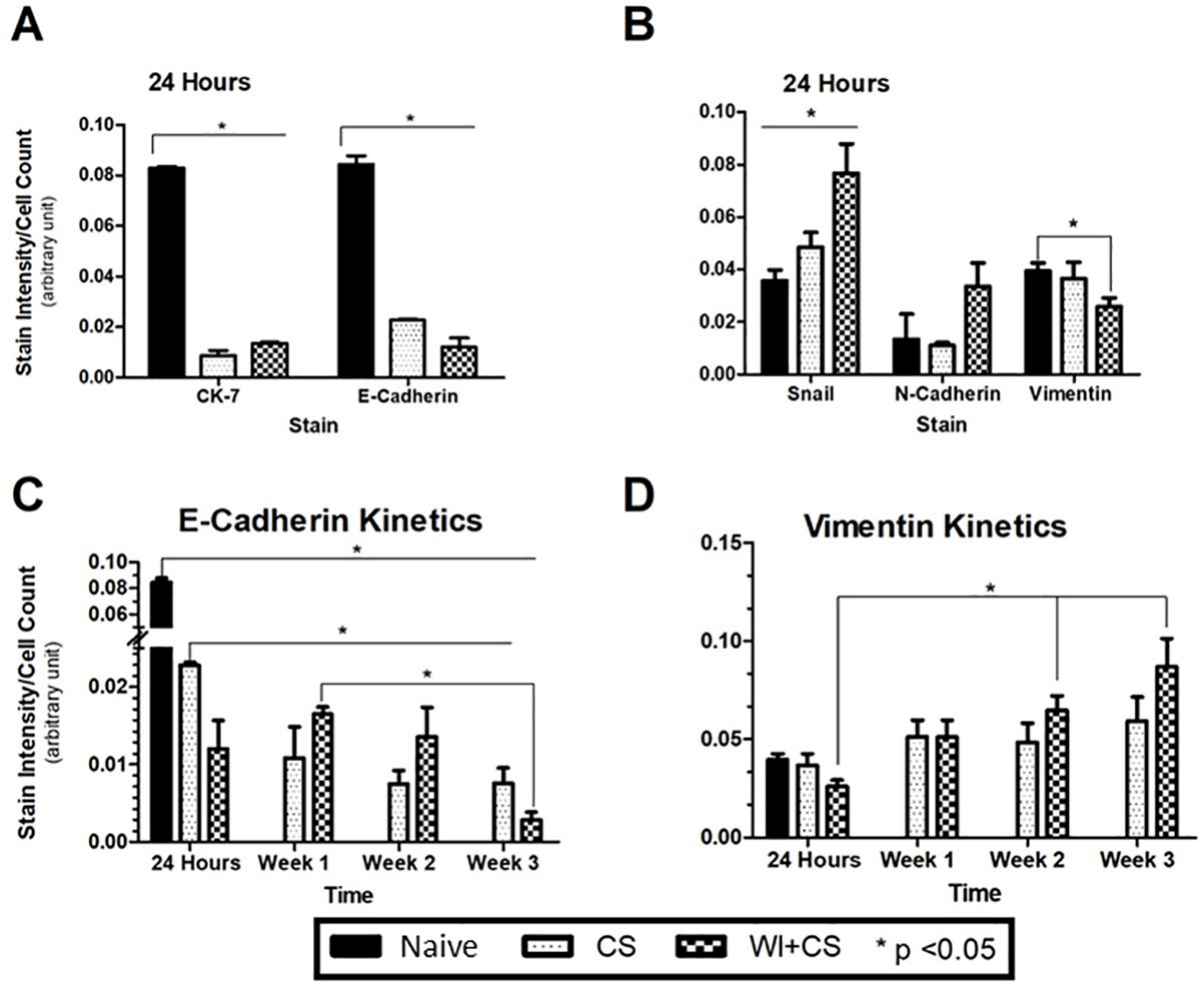
Epithelial protein expression profiles on cholangiocytes. Immunocytochemical expression of cholangiocyte CK7 and E-Cadherin (epithelial cell markers, panel A) and SNAIL, E-Cadherin, and Vimentin (mesenchymal markers, panel B) 24 hours after sham ischemia (Naive controls), after 24 hours after cold storage (CS), and after both warm ischemia (60 minutes) and CS (WI+CS). The kinetics of expression of E-Cadherin (Panel C) and Vimentin (Panel D) are also shown for all three groups of cholangiocytes. * P<0.05, data expressed as the sample mean +/- SD from 5 independent experiments.

Changes in protein expression profiles in cholangiocytes exposed to DCD ischemia conditions compared to fresh non-ischemic control cells are shown in **Fig 3** (representative fluorescent cell images) and **Fig 4** (curated data). Protein expression was measured using immunocytochemistry techniques. Early expression 24 hours after ischemia exposure for the epithelial-specific cell markers CK-7 and E-cadherin are shown in Fig 4 panel A while the mesenchymal-specific cell markers SNAIL, N-cadherin, and vimentin are shown in panel B. A kinetic pattern of expression in these cells is shown in Fig 4 panel C for E-cadherin and panel D for vimentin. Early expression of epithelial markers CK-7 and E-cadherin significantly fell after warm and cold ischemic exposure compared to non-exposed fresh cholangiocytes (Fig 4 Panel A) while the mesenchymal specific markers SNAIL, N-cadherin, and vimentin increase with DCD conditions, compared to fresh controls (Fig 4 panel B). Similarly, the loss of the epithelial specific marker E-cadherin increases as the time from the ischemic exposure increases (Fig 4 panel C) while the opposite happens for the expression of the mesenchymal specific marker vimentin (Fig 4 panel D). Values are mean ± SD from 4 independent experiments. These same trends can be visualized in the fluorescence intensity of the raw data images in Fig 3.

**Fig 5:**
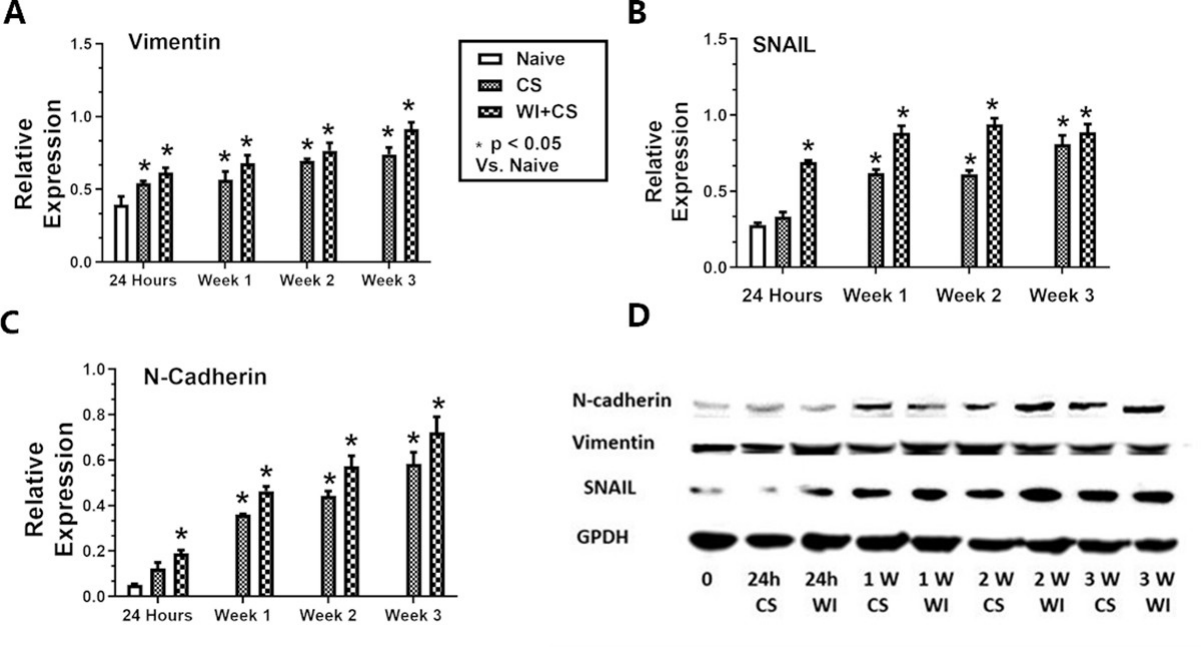
Mesenchymal protein expression profiles on cholangiocytes. Cholangiocyte expression (via western blot) of Vimentin (panel A), SNAIL (panel B), and N-Cadherin (panel-C) in Naive cholangiocytes, from those cold stored for 24 hours (CS), and from those with both 60 minutes of warm ischemia and 24 hours of cold storage (WI+CS). Expression is shown for 24 hours, 1, 2, and 3 weeks from ischemia exposure to staining. Panel D shows an actual chemiluminescent western blot analysis of these proteins and the GAPDH loading control. All samples were loaded at 30 μg protein per lane. *P<0.05, values are expressed as mean +/- SD from 5 independent experiments.

A western blot analysis of changes in mesenchymal specific cell markers (i.e. vimentin, SNAIL, and N-cadherin) over time in cholangiocytes isolated after exposure to DCD ischemic conditions is shown in **Fig 5** (panel D). The relative expression of these markers significantly increased in a time-dependent manner over the baseline values prior to ischemic exposure (panels A-C). Specifically, as the time from DCD exposure progresses from 24 hours to 3 weeks, the expression of these markers increased. Furthermore, there was a clearly higher expression response of these markers in the warm ischemic group, relative to cold ischemia alone (panels A-D).

**Fig 6:**
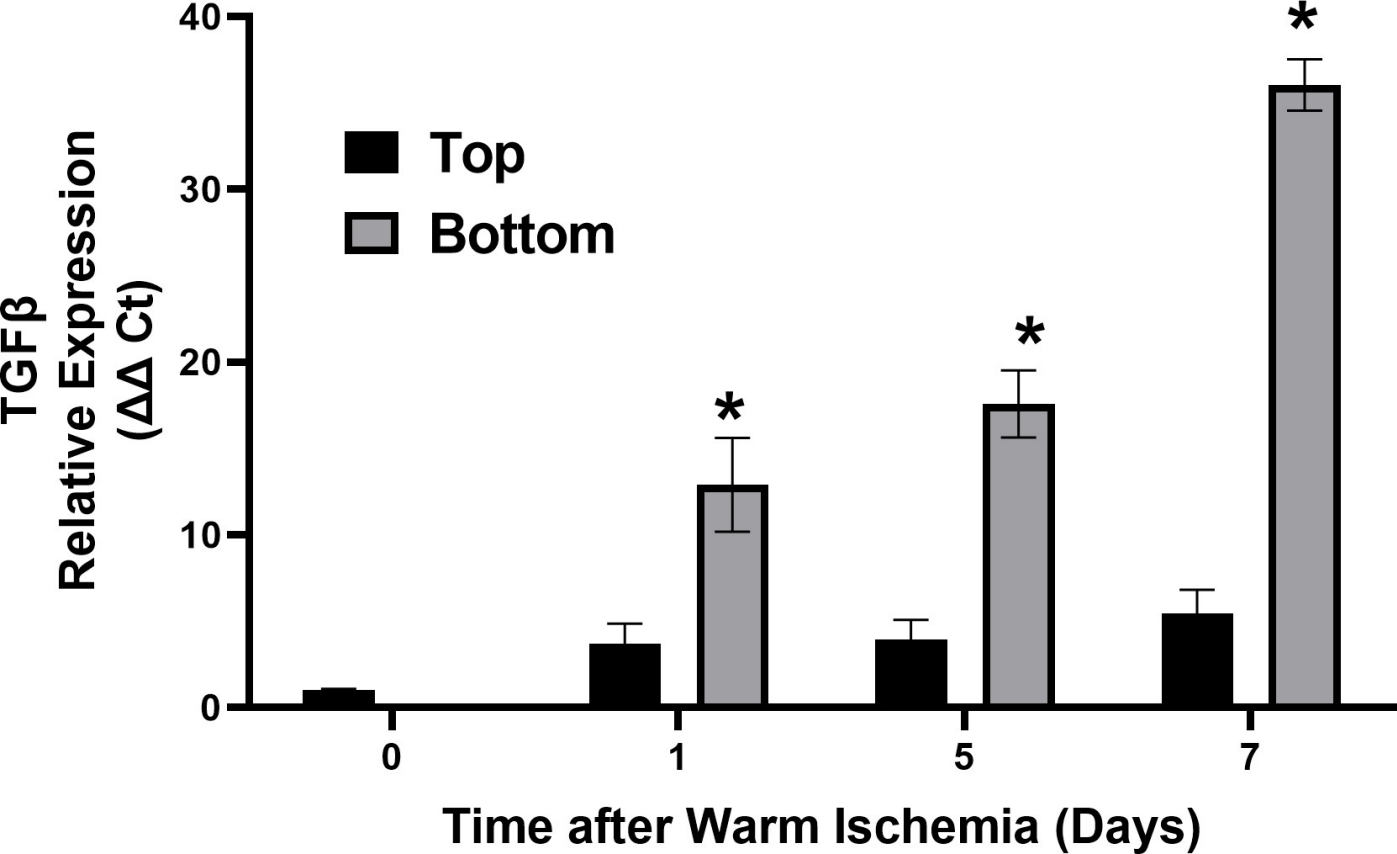
Cholangiocyte TGFβ gene expression and migratory behavior. qPCR results for TGF-β gene expression from DCD exposed cholangiocytes that either migrate (bottom) or do not migrate (top). The top and bottom are the respective top and bottom layer of cholangiocytes at the three time points (days 1, 5, and 7). The day 0 cells were cholangiocytes immediately after exposure to DCD ischemic conditions. Expression values are measured relative to the internal control GAPDH. * P< 0.05 relative to the corresponding top values, n = 4.

The differential expression of cholangiocyte TGF_β_ from cells exposed to DCD conditions that remained on the top of the migration chamber after 24 hours compared to cells that migrated through the chamber to the bottom are shown in **Fig 6**. Gene expression was measured by RT-PCR and shown as the relative expression from the GAPDH control gene (Δ Ct). DCD conditioned cells migrating to the bottom express multiple fold more TGF_β_ compared to DCD conditioned cells that don’t migrate (Top).

**Fig 7:**
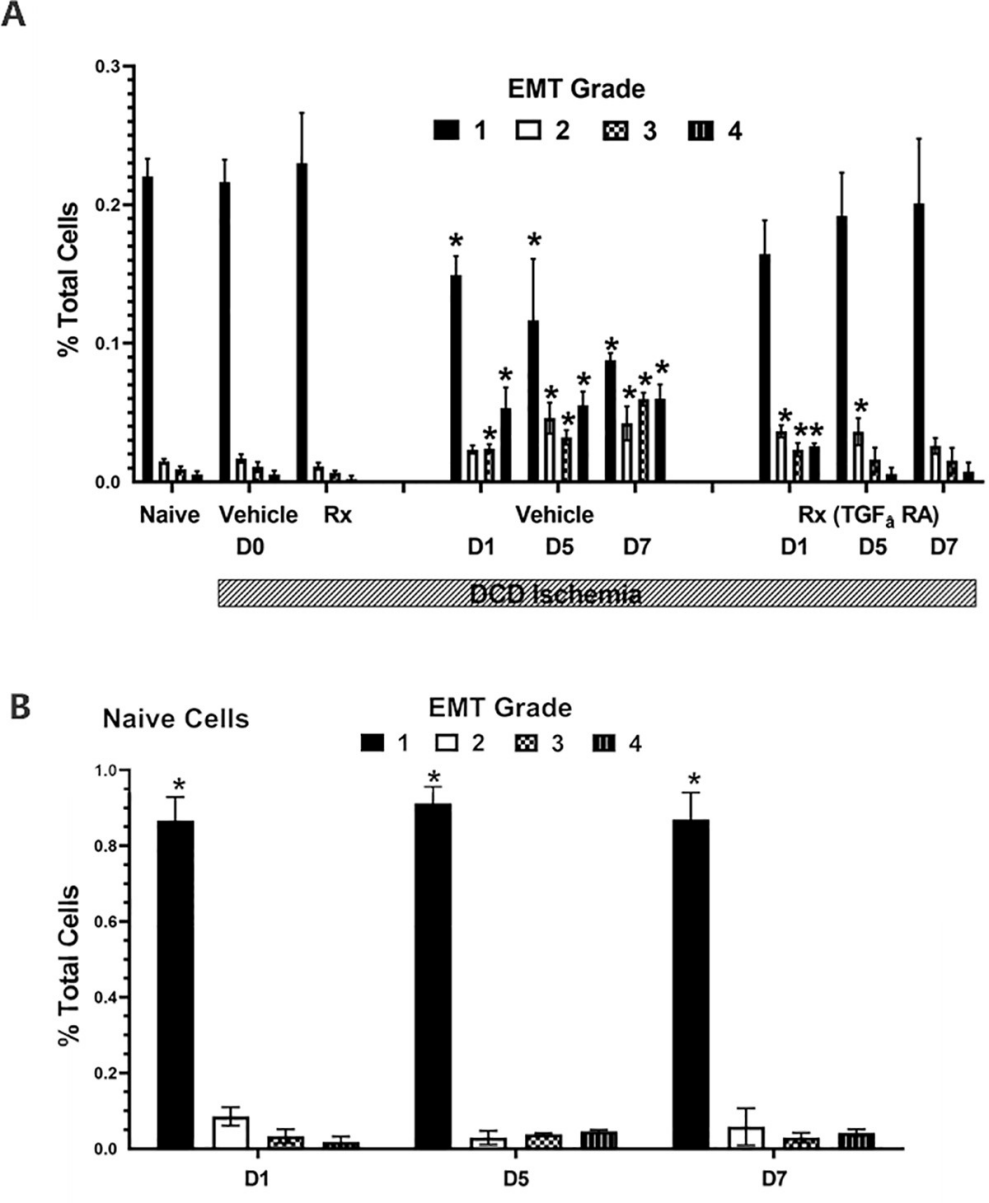
TGF_β_ signaling and cholangiocyte morphology changes. Cell morphology results from the grading of images taken with light microscopy. Data are the mean percent of cells out of total cells. There are three main groups, the fresh Naive cholangiocytes (Naive), vehicle DMSO control cholangiocytes, and cholangiocytes pretreated with the selective TGF_β_ receptor-1 antagonist Galunisertib (1 μM, 1 hour before DCD ischemia). Cells were examined for morphometric changes immediately after induction of DCD ischemia (D0), 1 day after (D1), 5 days after (D5), or 7 days after (D7). The bar indicates the groups exposed to DCD ischemic conditions of 1 hour of warm ischemia and 24 hours of cold storage followed by the indicated time in days of return to normoxia and 37C temperatures. G1 refers to normal cuboidal appearance, GII to mostly cuboidal appearance, GIII refers to mostly spindle appearance, and GIV refers to spindle appearance. All images were graded in a blinded manner. * P <0.05 relative to the corresponding bar in the control or treated (Rx) groups at D0. N = 4.

Human cholangiocyte morphology grades are shown in **Fig 7**. Most cholangiocyte cultures were exposed to DCD ischemic conditions and either pretreated with 0.01% DMSO in the culture media (Vehicle) or the selective TGF_β_ receptor-1 antagonist (RA) Galunisertib (Rx, 1 μM in DMSO) one hour prior to exposure to DCD conditions (Panel A). Then cells were immediately counted and scored for the degree of morphometric change at time 0 or placed into the tissue culture incubator and scored at days-1, -5, and -7 after exposure to DCD conditions. As a control, naïve cells (not exposed to DCD conditions) were plated and cultured for 7 days and their cell morphology was determined as before on days 1, 5, and 7 (Fig 7 panel B). These cells controlled for morphological changes that may be due to time dependent cell culture over 7 days and independent on DCD conditions. The morphological scores were based on the degree of change from purely cuboidal appearance characteristic of epithelium (Grade 1) to complete spindle shape characteristic of mesenchymal cells (Grade 4). Grade 2 cells were mostly cuboidal with some spindle shape while grade 3 were mostly spindle shape with some cuboidal appearance remaining. Naïve cells are cholangiocytes with no prior exposure to DCD conditions.

### Rat liver transplant model

**Fig 8:**
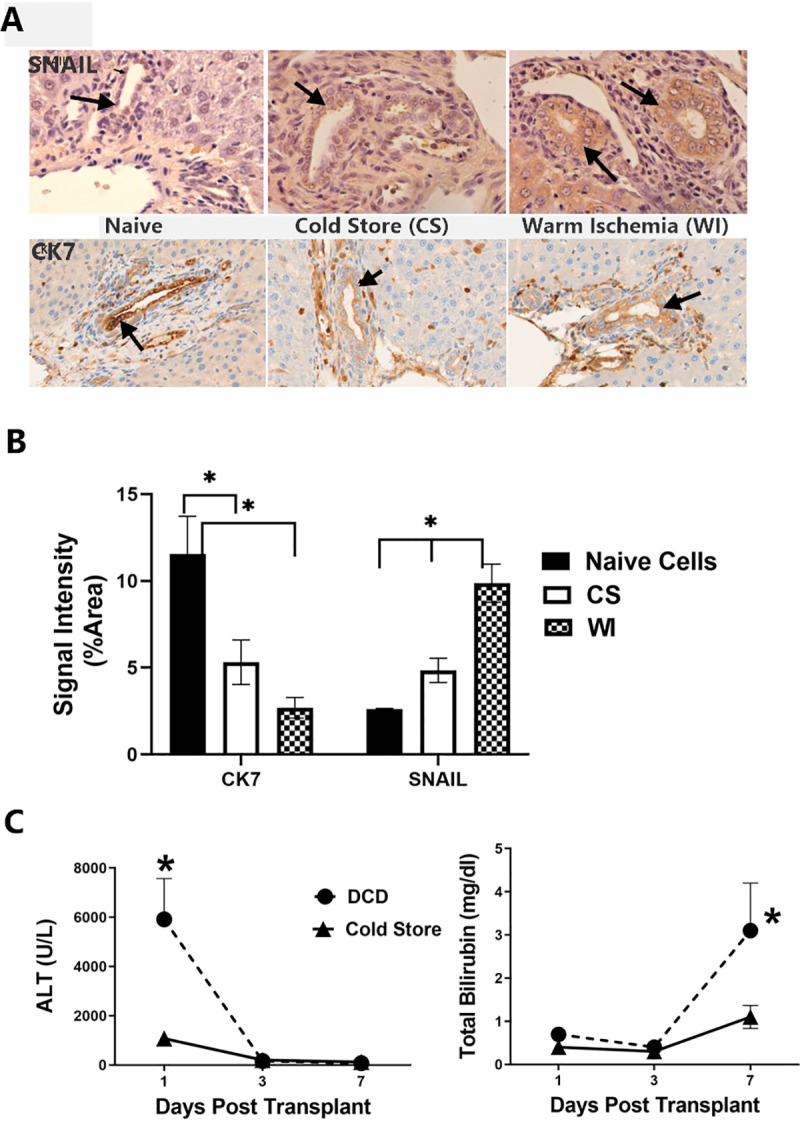
Rat liver ischemic cholangiopathy model. Immunohistochemical analysis of expression of SNAIL and CK-7 from rat liver tissue 7 days after transplantation into syngeneic rats. Sections were prepared from fresh non-ischemic livers, from livers with 24 hours of cold storage time in UW solution, and from livers with both 24 hours of cold storage and 30 minutes of warm ischemia prior to liver recovery from the donor (panel A). Arrows show individual cholangiocytes staining for the selected markers. Quantitation of stain intensity shown in panel A was done for each of the 5 livers from each group using ImageJ analysis. These results are graphically shown in Panel B, * P<0.05. Panel C shows the serum ALT and Total Bilirubin values measured in liver isograft recipients from the time of transplantation until 7 days after transplantation. Values are mean +/- SD from 5 rats, *P<0.05 relative to the control rats (24 hours of cold storage only).

Immunohistochemical detection of expression of the mesenchymal marker SNAIL and the epithelial marker CK-7 in fresh control liver tissue sections, sections from liver isografts with prior exposure to cold storage for 24 hours, and sections from liver isografts with prior exposure to warm ischemia for 30 minutes are shown in **Fig 8** (panel A). All liver isografts were recovered from the recipients 7 days after transplantation. The appearance of SNAIL expression is clearly seen in the biliary epithelial cells with liver grafts that were exposed to cold storage ischemia and more so with exposure to warm ischemia (panel A upper row). Conversely, CK-7 expression weakened significantly in biliary epithelial cells in sections from livers with prior exposure to DCD conditions (warm and cold ischemia, panel A-lower row). The signal intensity for these stains are shown in Fig 8B for CK7 and SNAIL in livers without DCD or transplantation (Naïve) and transplanted livers with cold storage for 24 hours, and with cold storage for 24 hours and with 30 minutes of preceding warm ischemia.

Finally, plasma transaminase (ALT) and total bilirubin values from rats with isografts exposed to DCD conditions Vs. sham surgery controls are shown in **Fig 8** (panel C). Transaminase levels rose dramatically on the first post-operative day in the DCD grafted recipients, compared to the controls but the levels normalized thereafter. Total bilirubin, however, stayed low in both groups until day 7 after transplantation, when it significantly spiked in the DCD group but not the control group, which were exposed only to cold storage for 24 hours.

## Discussion

Ischemic cholangiopathy following DCD liver transplantation is characterized by loss of biliary epithelium, loss of intrahepatic microscopic bile ducts, biliary constriction, and fibrosis. Although the molecular mechanisms of these phenotypic changes are unknown, it is hypothesized that the biliary epithelia first undergo an Epithelial to Mesenchymal Transition (EMT) that establishes the genetic reprogramming of the cells to transform them into cells with a mesenchymal phenotype. This is supported by data showing the loss of epithelialization and the establishment of fibroblast like cells with both smooth muscle and migratory behaviors in our cholangiocyte cell model of IC. In this model, we demonstrate the migratory behavior in the invasion assays, the smooth muscle appearance from the morphological assessments, and the genotypic shift from epithelial-specific to mesenchymal-specific gene expression. These EMT-specific cellular changes are correlated to exposure of these cells to DCD preservation conditions and establish a strong correlation between these two events. We further hypothesize that these events are causative in that DCD conditions cause both EMT and IC because we can prevent or reverse morphological EMT changes in human cholangiocytes pretreated with a specific inhibitor of TGF_β_ receptor-1, which is a canonical upstream activator of the EMT pathway in response to ischemic stress. What is lacking is evidence demonstrating detailed downstream signaling in cholangiocyte EMT, further causative evidence, and validation that EMT induced in human cholangiocyte cultures in response to DCD conditions can predict disease in human or animal models of ischemic cholangiopathy.

The hypothesis that EMT serves as a molecular mechanism of ischemic cholangiopathy is supported by data from this study and by similar events seen in kidney transplantation. Renal allografts can undergo chronic allograft nephropathy (CAN) or tubular atrophy with interstitial fibrosis (TA/IF) in some patients [22, 23]. This causes the kidneys to slowly fail months after transplantation independent from episodes of acute rejection. GFR and renal function slowly falls as the nephropathy develops over time. Chronic multiple injuries induced by preservation injury, donor co-morbidities, acute cellular rejection episodes, immune-mediated reactions, and calcineurin cytotoxicity may all cause additive injury that drives CAN [27]. Histologically, this condition is characterized by a drop out and loss of either renal tubular epithelium or entire tubules with the appearance of interstitial fibrosis [28]. This drop out of renal tubules leads to the drop in renal function over time. One of the strongest single risk factor for later development of CAN may be DCD preservation injury in the donor. This is known to cause an EMT in tubular epithelial cells, which may causally contribute to CAN. A parallel epithelial transformation may be occurring in the microscopic intrahepatic bile ducts because they are exposed to the same stress during DCD recovery and because the underlying attributes of the disease in the renal allografts is similar to that seen in ischemic cholangiopathy. Namely, these livers show loss of tubular epithelia, loss of whole bile ducts, smooth muscle like behavior in larger bile ducts, and interstitial fibrosis leading to liver and biliary failure. This is consistent with an EMT occurring in these epithelia. Perhaps multiple stress factors and insults experienced by DCD livers also produce additive or potentiating stimuli that finally drive the EMT switch. This is supported in our own data in these models by the accumulating effects of both prolonged cold storage and prior exposure to warm ischemia because both cause greater EMT outcomes than just cold storage alone. This is verified clinically in the IC rates in DCD livers where livers with prolonged hypothermia experience lower rates of ischemic cholangiopathy compared to livers with both prolonged cold storage exposure and a period of prior warm ischemia (DCD conditions).

The initiators, modifiers, and downstream pathways of EMT in this model are not known, but we suspect activation of TGFβ synthesis and receptor signaling is involved. In canonical EMT signaling, early synthesis of TGFβ_,_ followed by surface receptor ligation of the TGF_β_ receptor, and downstream SMAD signaling often initiates the EMT cascade. Other non-Smad signaling may also be involved. These could include stress activation of MAP kinase pathways such as p38, either dependent on upstream TGFβ signaling or independently through other upstream signaling through TNF_α_ or growth factors like Bone Morphogensis Proteins (BMP) and Hepatocyte Growth Factor (HGF) [29]. The p38 MAPK pathways may serve to modify the progression of a TGFβ-initiated EMT since p38 inhibitors can restrain the TGFβ response in epithelial cells [30]. Hepatocyte growth factor (HGF) may be another early initiator of EMT or serve to maintain the response initiated by TGFβ. HGF may be produced by existing mesenchymal cells already in the liver milieu, or from newly transformed cholangiocytes now assuming genetic and phenotypic mesenchymal properties. HGF binds to and activates c-MET, which are both expressed by human cholangiocytes [31].This signaling could serve to act as an early initiator of EMT by reducing the expression of e-cadherin, which is necessary for the cells to break apart to be able to later migrate. In fact, e-cadherin downregulation is observed in our transformed cholangiocytes after exposure to DCD conditions.

While the data involving TGFβ and its associated signaling components or modulators are not yet understood in our models, we know they are causally operational since TGFβ mRNA expression dramatically increases in human cholangiocytes after DCD conditions. Furthermore, the molecule is only expressed in the cells exposed to DCD conditions that also assume a migratory behavior, suggesting that TGF_β_ may have initiated the phenotypic shift in cytoskeletal protein reorganization towards an invasive phenotype. This is further supported by our ability to block these morphological changes in the exposed cholangiocyte by first pre-treating them with a selective TGFβ receptor-1 antagonist. These later data not only implicate autocrine TGFβ as an upstream initiator of EMT and IC-like behavior but supports the hypothesis that the descriptive changes in protein expression associated with EMT may be causatively involved in the IC phenotype. These data provide reassurance that if EMT in cholangiocytes exposed to DCD conditions significantly causes clinical IC in DCD liver allografts, then rational pharmacological manipulation of these initiator or downstream circuits could prevent or reverse clinical disease. The role of other early initiators in the EMT cascade in DCD exposed cholangiocytes such as BMP and HGF or modifiers like HDAC or HIF_1α_ also need to be explored. These pathways may represent attractive targets to either prevent or arrest an existing EMT and IC since small molecule biologically active and stable drug candidates already exist for these targets. That is the ultimate goal in studying these pathways.

The EMT changes observed in our primary human cholangiocyte model of DCD preservation injury support the hypothesis that EMT is a causal factor in the development of ischemic cholangiopathy in human liver allografts recovered from DCD donors. This is supported by preliminary data obtained from our early rat liver transplant model. In these studies, we observed that rat liver isografts exposed to 30 min of prior warm ischemia before cold storage began to show signs of biliary complications seven days after transplantation, compared to rat liver isografts without the prior exposure to warm ischemia. This is based on the rapid spike in total bilirubin values measured in the plasma of the recipient rats at day 7 after transplantation compared to the normal baseline values seen in the first 6 days after transplantation or in the rats with just exposure to cold storage. This suggests that something changed in the DCD livers that was causing complications in the normal handling of bile acids in the liver, which is an early clinical feature of IC in patients with DCD liver grafts. Furthermore, immunohistochemical analysis of these tissues in the two groups of rat liver isografts indicated an increased expression of SNAIL in the cholangiocytes in DCD livers, relative to livers with only cold storage. Conversely, we observed a decrease in the expression of the epithelial cell marker CK-7 in the DCD livers over time compared to livers with only cold storage. These early changes in gene expression in the DCD liver isografts at the time we observed biliary changes is consistent with the hypothesis that these livers were in the early stages of IC and that this may have been caused by the pre-existing exposure to warm ischemia at the time of liver recovery. To test this hypothesis directly, future studies will pharmacologically interfere with early EMT signaling after transplantation to see the effect this has on liver function and differential gene expression at longer reperfusion times.

In conclusion, primary human cholangiocytes exposed to warm ischemia and cold storage displayed morphologic, genetic, and phenotypic changes consistent with EMT. The expression of TGFβ is involved in morphological changes characteristic of IC. The EMT signaling pathway may be a targetable mechanism to treat post-transplant biliary complications in DCD liver allografts or to prevent IC from occurring by acting in the organ recovery or preservation period.

## Supporting information

S1 File Immunocytochemical image data for Figs 2 and 3. (PPTX)

S2 File Data for Fig 4. (PZF)

S3 File Data for Fig 5. (PZF)

S4 File Data for Fig 6. (PZFX)

S5 File Data for Fig 7. (XLSX)

S6 File Data for Fig 7A. (PZFX)

S7 File Data for Fig 8B. (PZFX)

S8 File Data for Fig 8C. (PZF)
