# Supplementary material for: Cholangiocyte Epithelial to Mesenchymal Transition (EMT) is a potential molecular mechanism driving ischemic cholangiopathy in liver transplantation
Source: PLoS One. 2021 Jul 7;16(7):e0246978. doi: 10.1371/journal.pone.0246978 (PMC8263302; doi:10.1371/journal.pone.0246978)

## Slide 1
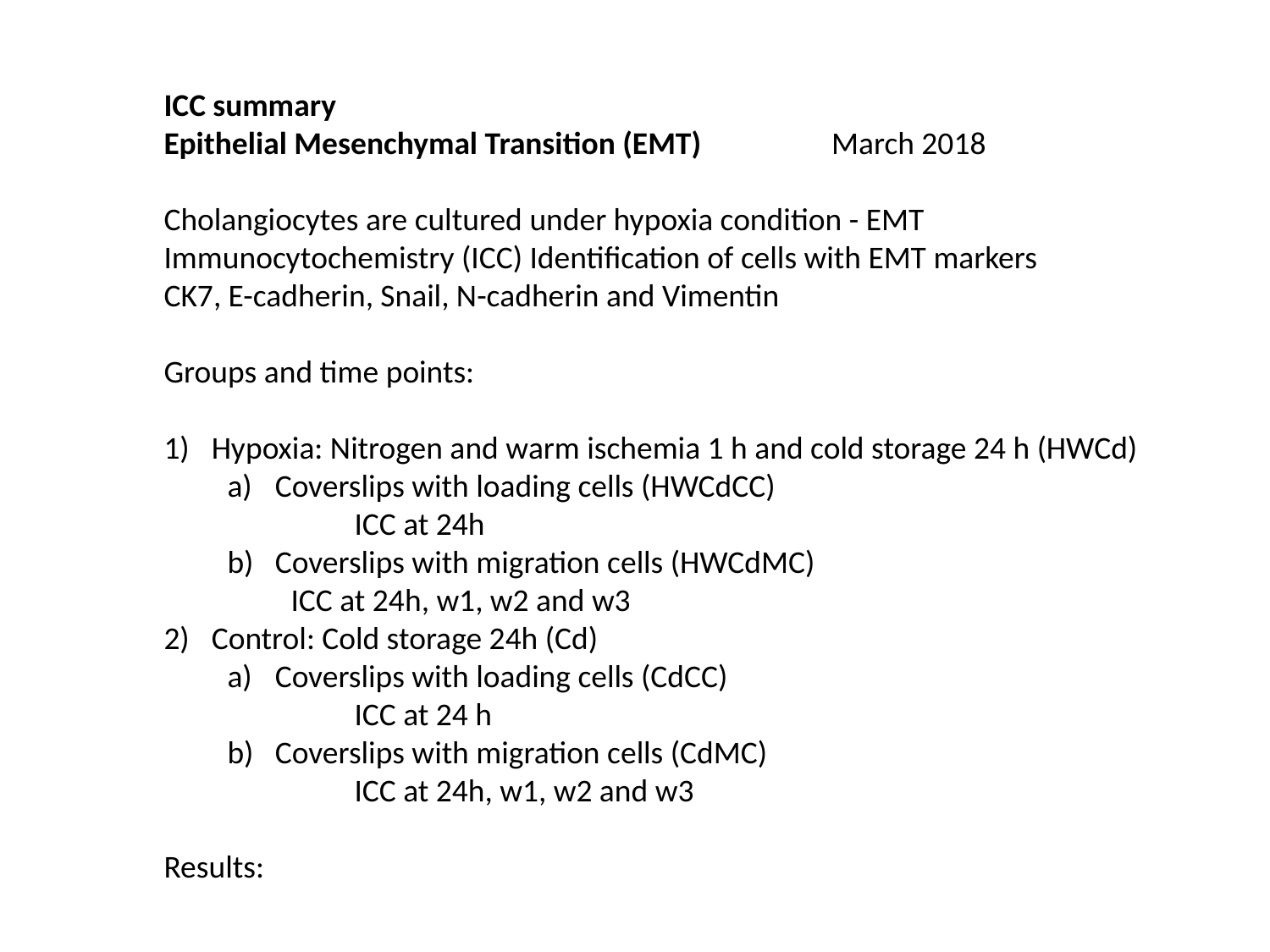

ICC summary
Epithelial Mesenchymal Transition (EMT) March 2018
Cholangiocytes are cultured under hypoxia condition - EMT
Immunocytochemistry (ICC) Identification of cells with EMT markers
CK7, E-cadherin, Snail, N-cadherin and Vimentin
Groups and time points:
Hypoxia: Nitrogen and warm ischemia 1 h and cold storage 24 h (HWCd)
Coverslips with loading cells (HWCdCC)
	ICC at 24h
b) Coverslips with migration cells (HWCdMC)
ICC at 24h, w1, w2 and w3
Control: Cold storage 24h (Cd)
Coverslips with loading cells (CdCC)
	ICC at 24 h
b) Coverslips with migration cells (CdMC)
	ICC at 24h, w1, w2 and w3
Results:

## Slide 2
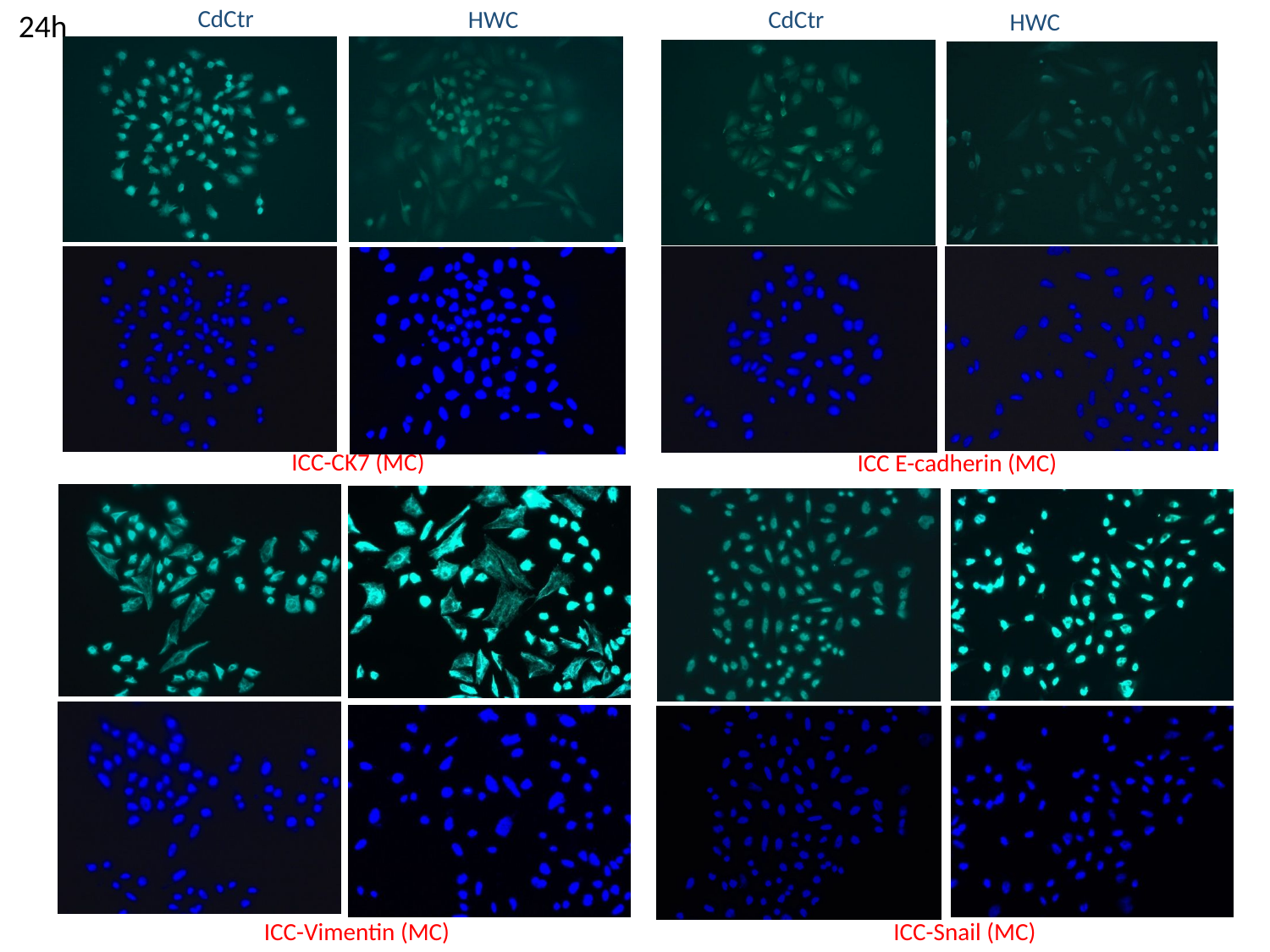

24h
HWC
CdCtr
HWC
CdCtr
ICC-CK7 (MC)
ICC E-cadherin (MC)
ICC-Vimentin (MC)
ICC-Snail (MC)

## Slide 3
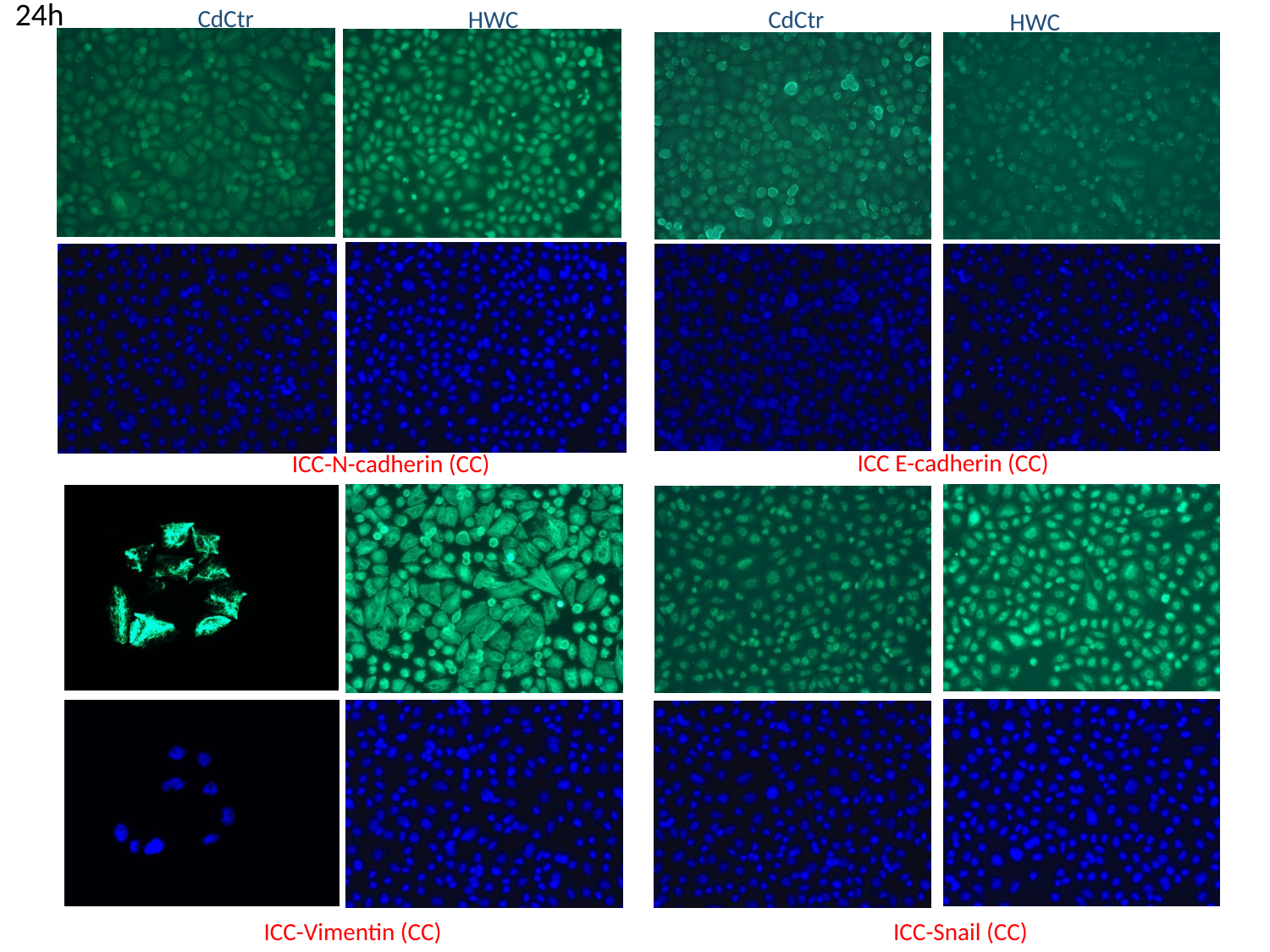

HWC
24h
CdCtr
HWC
CdCtr
ICC E-cadherin (CC)
ICC-N-cadherin (CC)
ICC-Vimentin (CC)
ICC-Snail (CC)

## Slide 4
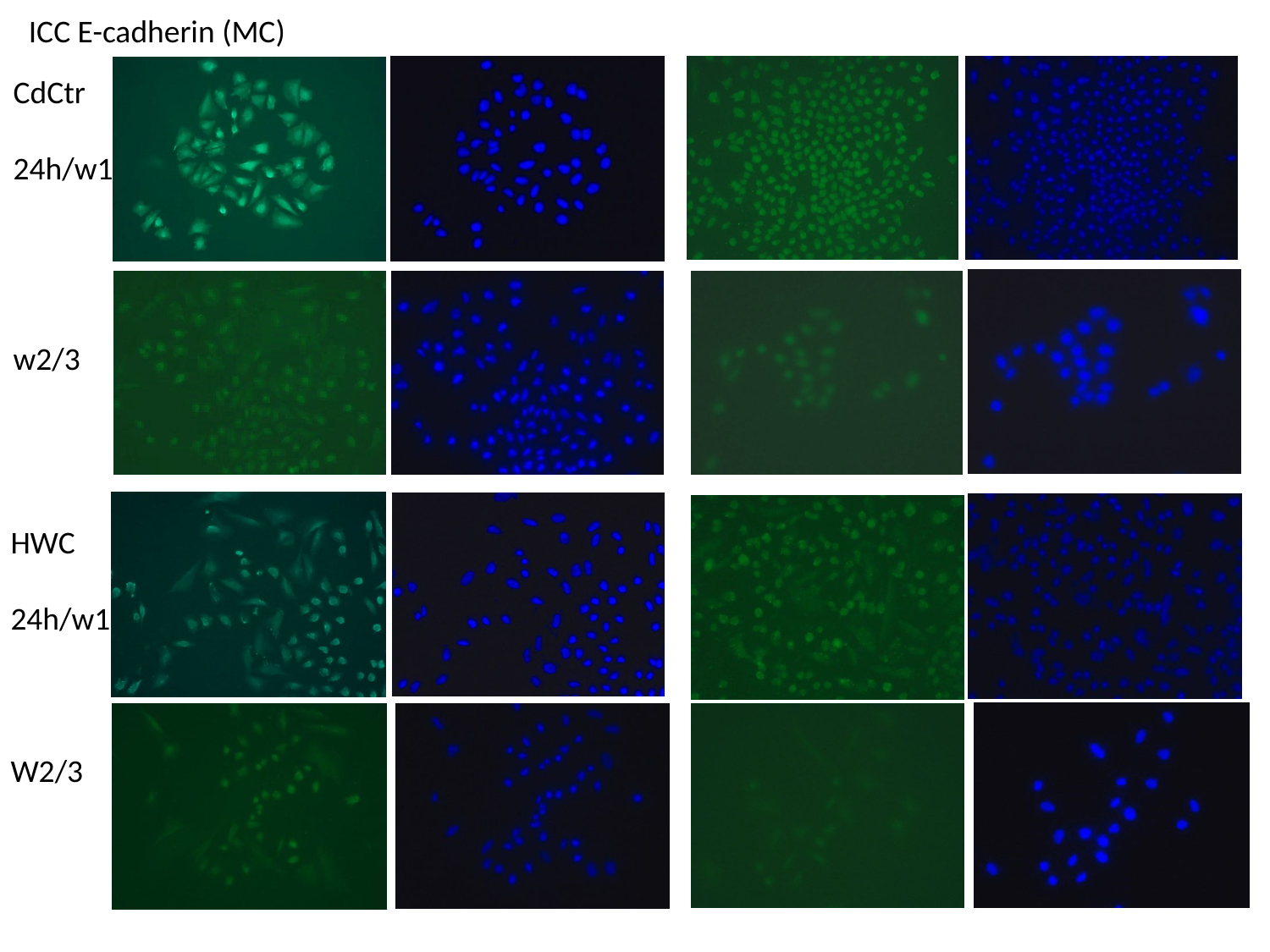

ICC E-cadherin (MC)
CdCtr
24h/w1
w2/3
HWC
24h/w1
W2/3

## Slide 5
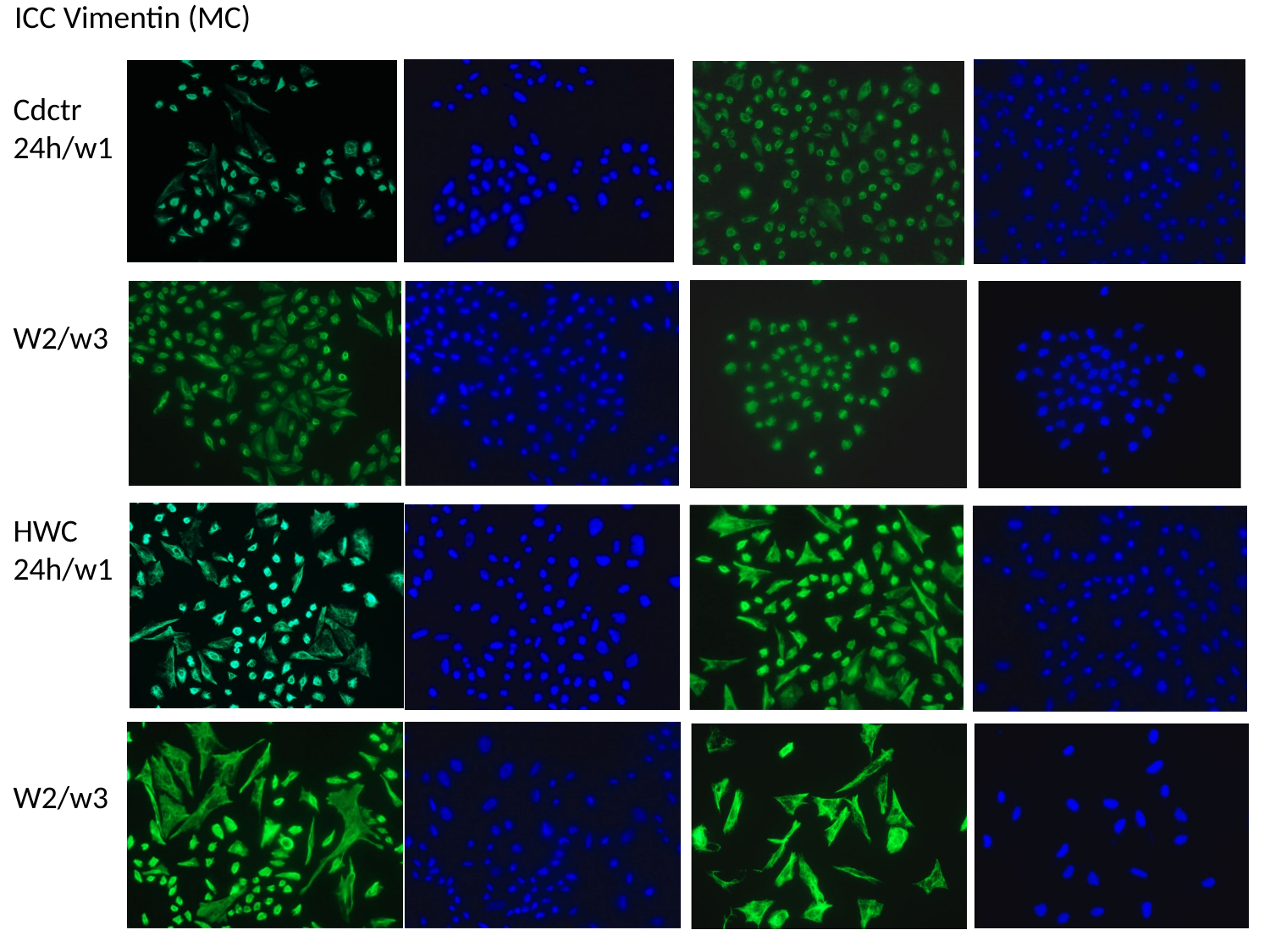

ICC Vimentin (MC)
Cdctr
24h/w1
W2/w3
HWC
24h/w1
W2/w3

## Slide 6
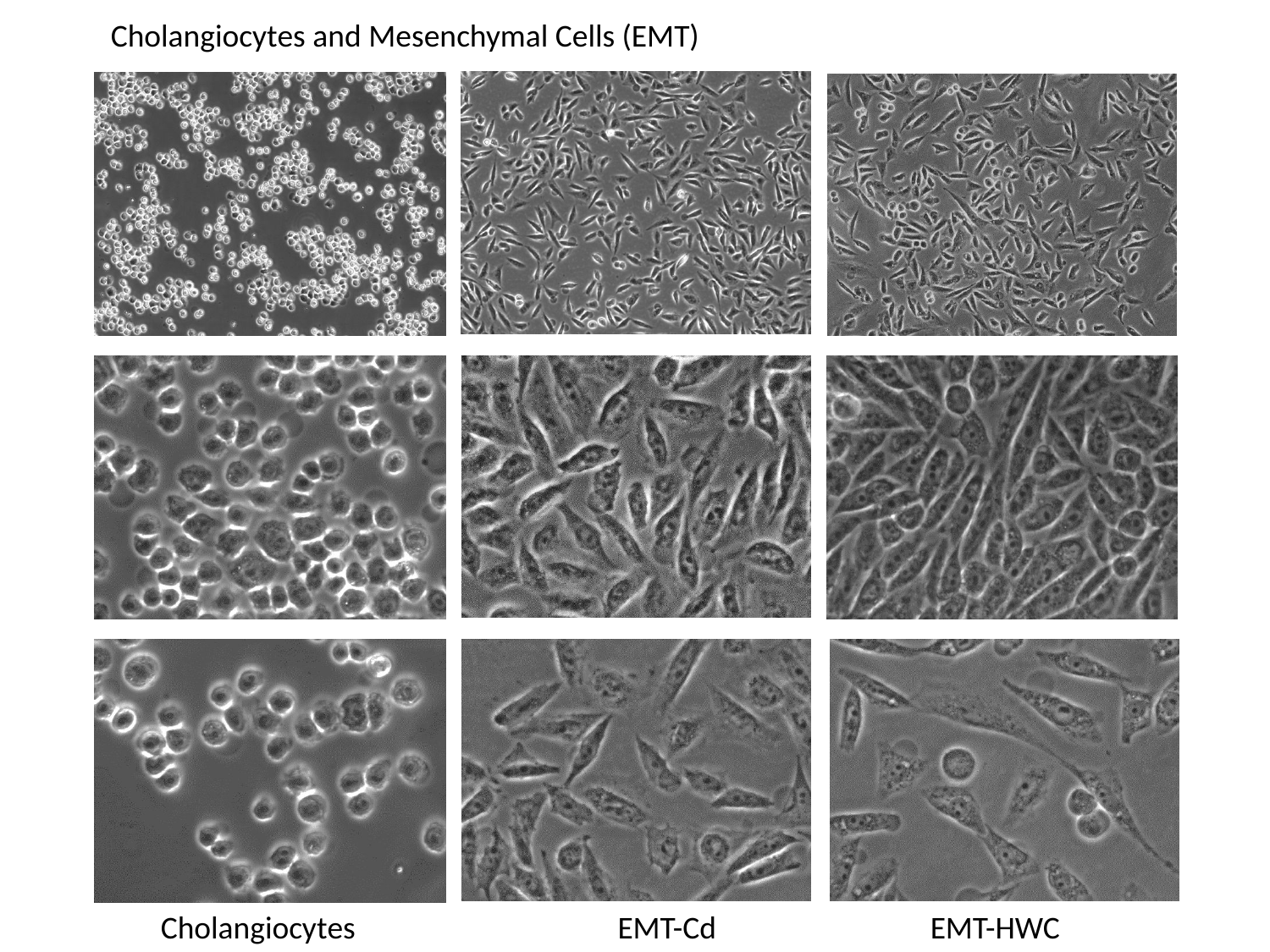

Cholangiocytes and Mesenchymal Cells (EMT)
Cholangiocytes
EMT-Cd
EMT-HWC

## Slide 7
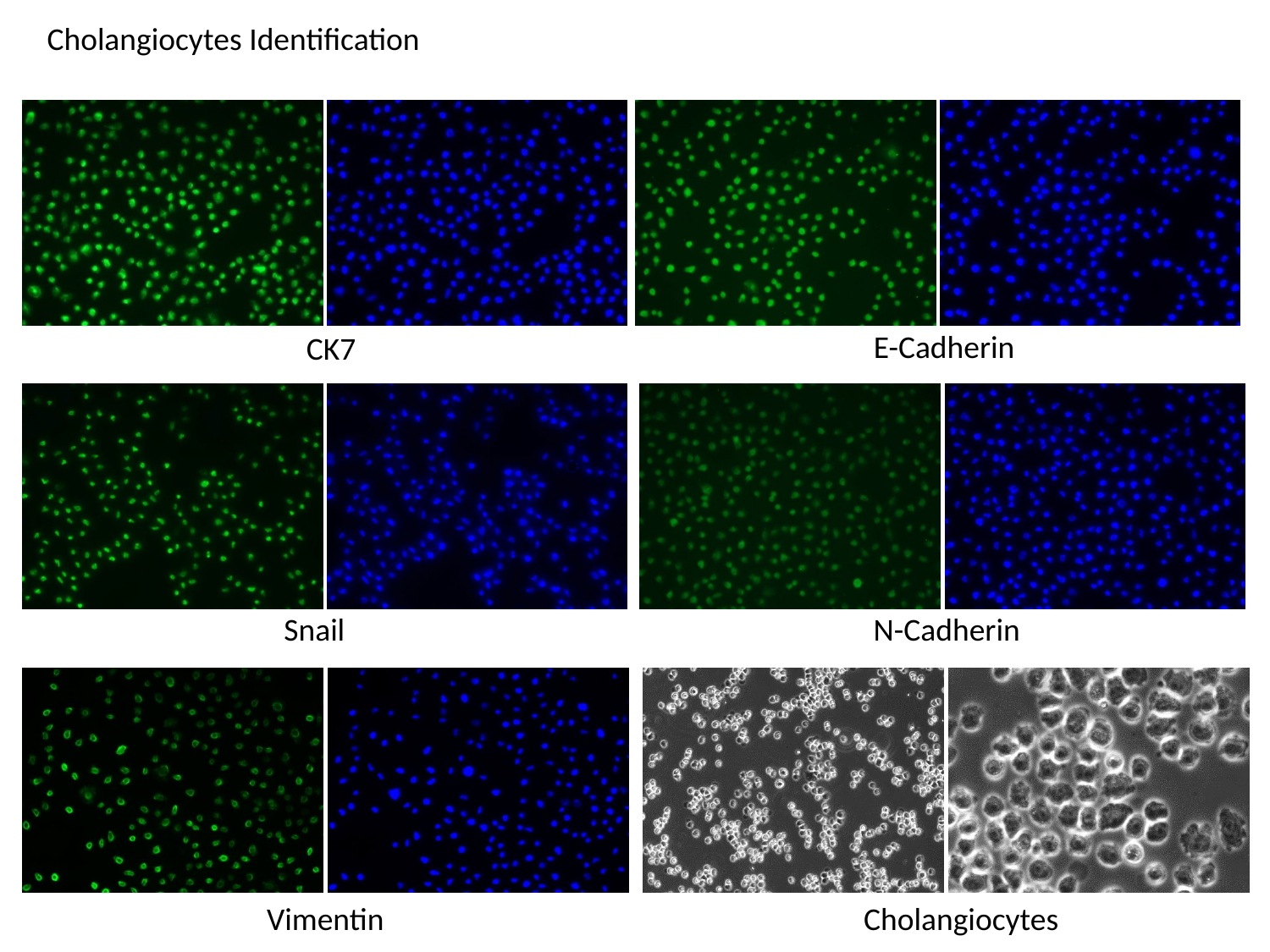

Cholangiocytes Identification
E-Cadherin
CK7
N-Cadherin
Snail
Vimentin
Cholangiocytes

## Slide 8
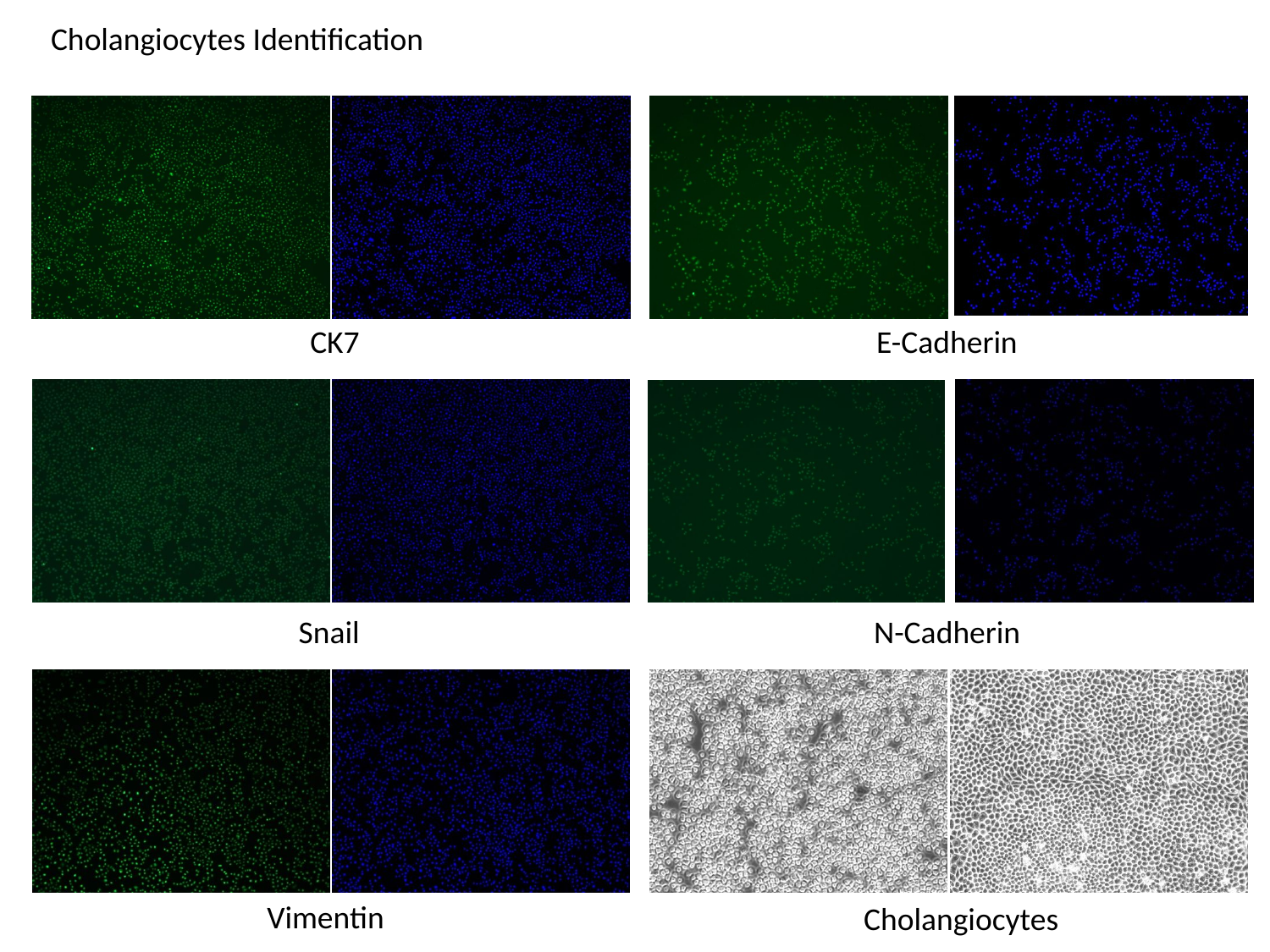

Cholangiocytes Identification
CK7
E-Cadherin
Snail
N-Cadherin
Vimentin
Cholangiocytes

## Slide 9
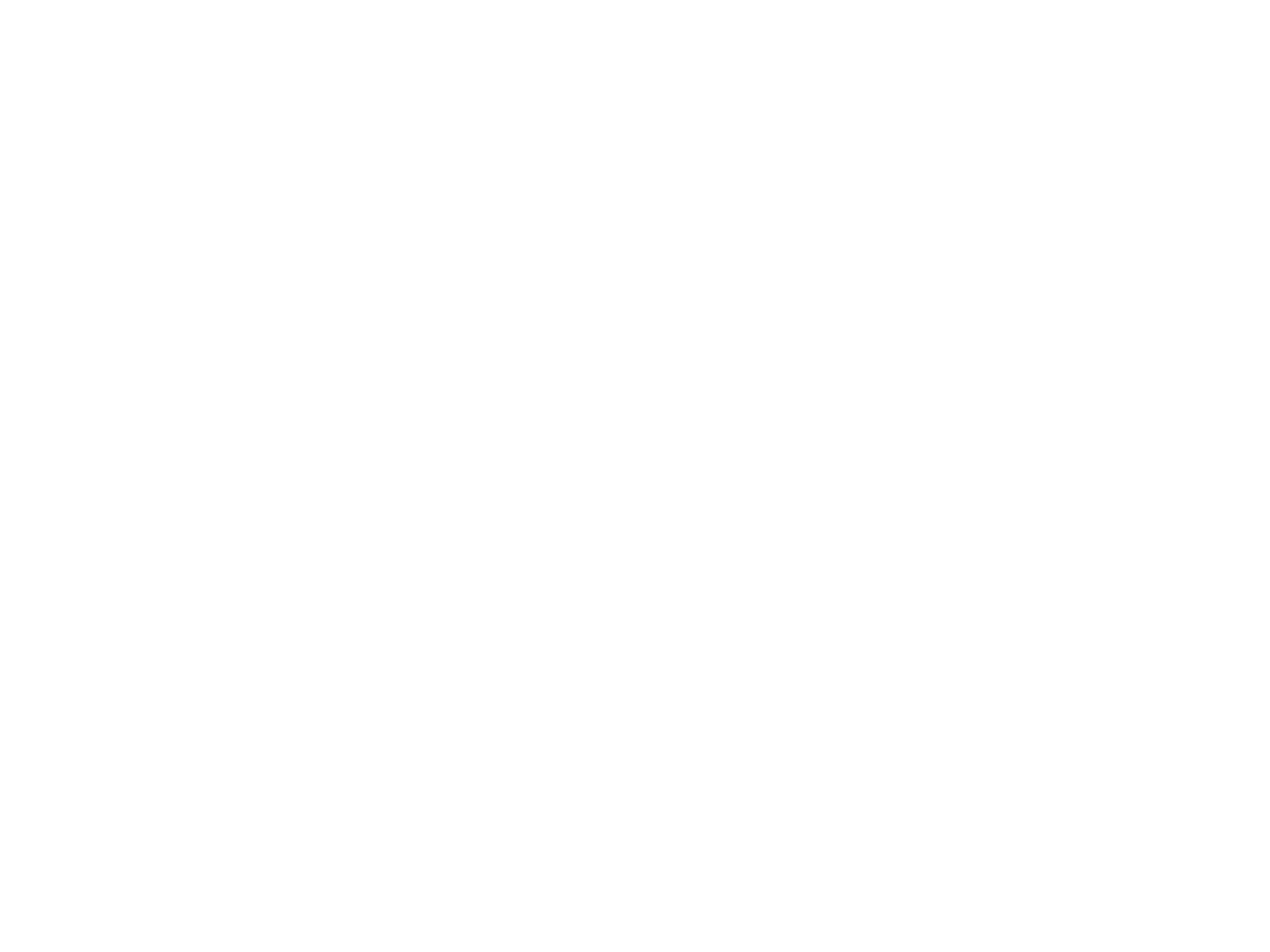

Supplement: S1 File — (PPTX) [file pone.0246978.s001.pptx]
